# Sex‐specific genetic differences in endurance swimming of Trinidadian guppies

**DOI:** 10.1002/ece3.1789

**Published:** 2015-10-28

**Authors:** Swanne P. Gordon, Yun Yi Chen, Karalynn Yamashita, Christopher Bejar, Adam Wilshire, Vinson Cheung

**Affiliations:** ^1^ Centre of Excellence in Biological Interactions Department of Biological and Environmental Sciences University of Jyväskylä Jyväskylä Finland; ^2^ Department of Biology University of California Riverside California

**Keywords:** Common‐garden experiment, critical swimming speed, fish, gender‐specific effects, locomotive adaptation, predation

## Abstract

Swim performance is considered a main fitness‐determining trait in many aquatic organisms. Swimming is generally the only way most aquatic prey can escape predation, and swimming capacity is directly linked to food capture, habitat shifts, and reproduction. Therefore, evolutionary studies of swim performance are important to understand adaptation to aquatic environments. Most studies, however, concentrate on the importance of burst‐swim responses to predators, and little is known about its effect on endurance. Even fewer studies associate differences in organism swim capabilities to key gender‐specific responses. In this experiment, we assess the gender‐specific genetic basis of swimming endurance among four different populations of Trinidadian guppies adapted to different predation regimes. Our results show that second‐generation common‐garden females adapted to a low‐predation environment show longer swim endurance than fish adapted to a high‐predation environment. We also find an expected effect of lowered swimming endurance during pregnancy, but interestingly, it did not matter whether the females were in advanced stages of pregnancy, which severely changes body morphology, versus mid‐pregnancy. Males did not show the same trends across populations, and overall had lower swim endurances than female fish combined even when accounting for size differences. Populations recently transplanted from high‐ to low‐predation environments showed similar endurance to natural low‐predation environments in one population but not the other. This study highlights the importance of endurance in the adaptation of aquatic organisms to different predation regimes.

## Introduction

Locomotive adaptation in response to environmental factors has long been a research interest in many taxa and disciplines (Domenici and Blake 1997; McGuigan et al. 2003; Fulton et al. 2005; Higham 2007; Marras et al. 2011). From an evolutionary standpoint, the interest lies in understanding how heritable variation in traits that affects locomotion responds to natural selection. Because locomotion is a strong determinant of an individual's interaction with their environment – be it foraging, predator escape, or mate searching – it is particularly important to our understanding of whole‐organism adaptation (phenotypic integration sensu, Pigliucci 2003), and the selective trade‐offs involved. Despite this, studies examining how shifts in selection regime affect the evolution of whole‐organism performance traits in the wild, such as locomotive mobility, are rare (Dalziel et al. 2011).

Maximizing mobility while minimizing energetic costs in wild aqueous environments has typically produced three categories of swimming performance in fish: sustained, prolonged, and burst speed swimming. Although the literature may differ slightly on ranges of each category, sustained swimming is generally performed using mainly red myotomal muscle, which enables fish to maintain constant speed locomotion for extended periods of time (>200 min) with little fatigue (Beamish 1978; Webb 1994; Breen et al. 2004). This swim type is most likely important in long‐distance movement or migration. Prolonged swimming includes the use of more white myotomal muscle and the added use of multiple muscle functions that decrease endurance but increase speed (Breen et al. 2004). This strategy (usually ranging from 20 sec to 200 min) is generally employed during medium‐distance movement when searching for food or mating partners, and holding stationary amidst heavy current (Blake 2004). Burst swimming on the other hand refers to the fastest performance (<20 sec) that fish can do, and use mainly white myotomal muscles that have high power outputs (Webb 1994). It usually describes the more complex locomotor movements exhibited by fish while changing direction or velocity in the water. This form of swimming is commonly observed during predator evasion, capturing evasive prey, and social interaction (Langerhans and Reznick 2010). For example, fish that are adapted to environments where they are at risk of predation generally become faster at evading predators than fish from predator‐free environments.

Even with all the research carried out on locomotive adaptation, there still remain some important gaps in our knowledge. First, many systems focus only on rapid escape ability or burst swimming, rather than other swimming types, which themselves may also be subject to selection. This is unfortunate because the ability to escape predators (or capture prey) by accelerating at a faster rate (burst swimming) generally comes with a trade‐off in endurance swimming (sustained or prolonged swimming) ability, which may be more effective for nutrient foraging in predator‐free sites (Weihs 1973; Webb 1984; Domenici 2003; Blake 2004; Langerhans 2009). For example, Oufiero et al. (2011) found that Hart's killifish (*Rivulus hartii*) showed a negative relationship between critical swimming speeds and sprint speeds: High‐predation killifish were faster sprinters but then had lower endurance than killifish from low‐predation sites. As a result, shifts in predation levels could influence changes in different types of swimming performance due to changes in selection pressures.

Second, many systems focus on swim performance in one sex, while ignoring the other. This should also not be the case, because decreases in natural selection (e.g., via predation) are often connected to a relative strengthening of sexual selection. Shifts in the relative importance of natural versus sexual selection are likely to have different impacts in the two sexes and could lead to the manifestation of gender‐associated differences in swimming performance. An example of this could be nicely explored in systems such as guppies. Trinidadian wild guppies are typically divided into two ecotypes: high versus low predation. High‐predation populations are usually found in the downstream reaches of rivers, where they coexist with predatory fish that have strong effects on guppy demographics. Low‐predation populations on the other hand are typically found in upstream tributaries above barrier waterfalls, where the predation risk is low or relatively absent (Gordon et al. 2009), and water velocity is higher (Marshall et al. 2012). Instead, in low‐predation environments, sexual selection via female choice for highly colorful males plays the seemingly stronger role (Houde 1997). Male guppies in high‐predation environments may hence be selected for higher degrees of burst swim abilities, perhaps leading to subsequently lower endurance swimming. Low‐predation male guppies may otherwise benefit more from higher degrees of endurance swimming in their mainly predator‐free sites. This, because many species of male poeciliids, such as guppies (*Poecilia reticulata*) or even swordfish (*Xiphophorus* spp.), will court females with elaborate sexual displays (enhanced in low‐predation environments where there is less risk of predators) where an increased capability of prolonged swimming or endurance may give them a fitness advantage to be able to court for longer periods of time (Bisazza 1993; Basolo and Alcaraz‐Zubeldia 2003; Oufiero 2011). Similar selective pressures in swimming capabilities (efficient rapid escape of predators), on the other hand, may drive female guppies in high‐predation environments. However in low‐predation environments where sexual selection is again stronger, they may have completely different responses in endurance swimming where they may be more concerned with foraging in their generally higher velocity environments typical of low‐predation streams (Marshall et al. 2012), and evading increased male courting. In this respect, we may expect that gender differences in swimming capabilities in guppies would be larger in low‐predation sites compared to high‐predation ones.

**Figure 1:**
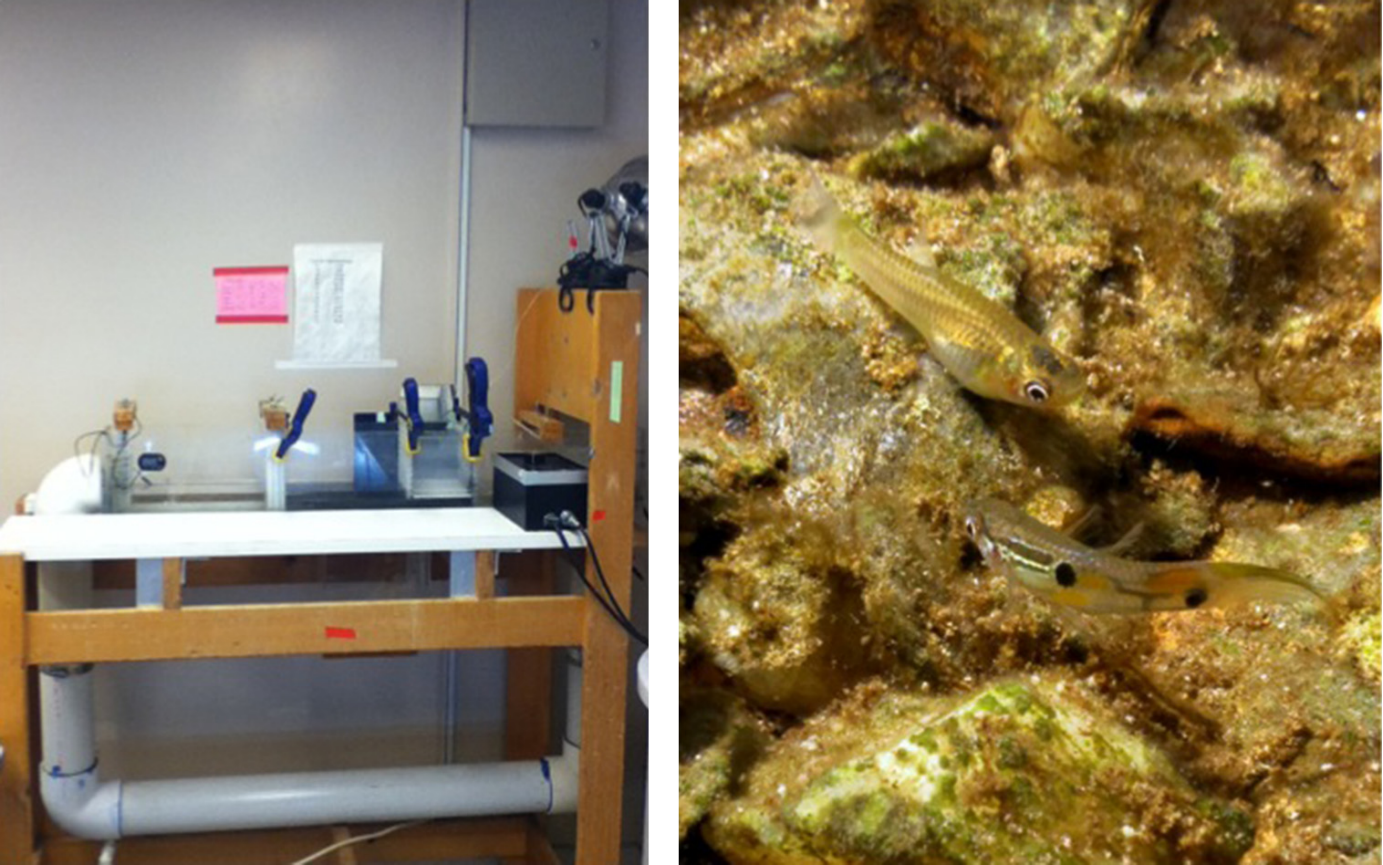
Picture of swim tunnel and female versus male guppy.

Third and final, research in locomotive adaptation rarely examines whether observed differences in swimming ability bear a strong genetic signature. This is important, because locomotor ability and related traits often display a high degree of plasticity (Huntingford et al. 1994; O'Steen et al. 2002 *;* Aubret et al. 2007), and evolution can only respond to genetic variation. In this study, we evaluate the genetic basis of endurance (via prolonged swimming performance) in male and female populations of high‐ and low‐predation‐adapted Trinidadian guppies (*Poecilia reticulata*) (Fig. 1). Since swimming performance is influenced by both changes in body morphology and an increasing energetic burden in pregnant female fish (Plaut 2002; Svendsen et al. 2013), we also examine, for the first time to our knowledge, the effect of pregnancy on endurance (sustained or prolonged) swimming in guppies. We hypothesize that carrying offspring (which causes a distension of the female form) negatively impacts endurance swimming.

### Guppy system and swimming

Adaptation to changes in predation has driven the evolution of various life history and morphological differences between the two ecotypes of guppies: high and low predation. Molecular studies show that all low‐predation guppy populations were founded from high‐predation populations that colonized upstream portions of each river basin (Alexander et al. 2006; Suk and Neff 2009). This means that every low‐predation population represents an independent shift in selection regime from ancestral high‐predation phenotypes. High‐predation guppies are smaller in size, mature earlier, have deeper and longer caudal peduncles, and more shallow bodies than their counterpart low‐predation guppies (Reznick et al. 1996; Nicoletto and Kodric‐Brown 1999; Langerhans and DeWitt 2004; Hendry et al. 2006). Given these morphological differences, we would expect a difference in swimming performance. Indeed, prior research has linked swimming performance to body shape and size in a variety of organisms (Domenici 2003; Langerhans 2009). Endurance swimming is usually associated with a more streamlined body shape, narrow caudal peduncle, high aspect ratio (caudal fin height compared to its surface area), and large body depth (Webb 1984). Burst swimming, on the other hand, is associated with larger caudal fins, deeper bodies, and deeper caudal peduncles (Webb 1984; Langerhans et al. 2004; Domenici et al. 2008).

Previous research on guppy predator‐driven swimming differences has typically focused on rapid escape ability or burst swimming. High‐predation guppies are faster and better at maneuvering than guppies from low‐predation localities (O'Steen et al. 2002; Ghalambor et al. 2003). These studies have been performed using fish from both natural and common‐garden‐reared environments and hence suggest these changes have a genetic basis. Only one study thus far has paid attention to the alternative strategy, endurance swimming, in different predation regimes. This study, focused exclusively on males, showed to a varying degree that low‐predation populations have higher swimming endurance performance than high‐predation ones (Nicoletto and Kodric‐Brown 1999). In this study, however, these differences were measured on wild rather than common‐garden fish, making it impossible to assess a genetic basis to the trait.

### Research questions

In this study, we test for gender‐specific genetic differences in the swim endurance of Trinidadian guppy populations adapted to different predation regimes. We do so by comparing the endurance swim of second‐generation common‐garden individuals (both males and females) originating from four guppy populations. Rearing the fish under common conditions removes environmental effects experienced by the wild‐caught juveniles before capture and suggests that any changes between populations have a genetic basis. Two of the four populations used in this study represent recent translocations of natural high‐predation fish into two low‐predation environments. Given that all low‐predation populations naturally descend from high‐predation populations (Alexander et al. 2006), we can distinguish the influence of recent predation history (translocation) and historical adaptation to different environments (natural divergence). Considering that these populations have already evolved key fitness‐related traits (such as body size; Gordon et al. 2015) which have been shown to affect swimming capabilities, it will be interesting to test whether it has already led to evolutionarily divergent differences between ancestral and introduced fish. We specifically answer three questions: (1) Does sex and pregnancy status affect guppy endurance swimming? (2) Are there genetic differences in endurance swimming between high‐ and low‐predation adapted populations? and (3) Are these habitat‐associated differences sex‐specific?

## Methods

### Study populations and rearing

This study uses second‐generation fish reared in our laboratory at the University of California, Riverside. In February 2009, juvenile guppies were collected from four wild populations on the Guanapo River in the island of Trinidad in the Southern Caribbean. These included fish from a natural high‐predation population (GH), a natural low‐predation population (GL), and two transplanted populations into low‐predation tributaries (Upper Lalaja, UPL; and Lower Lalaja, LOL). UPL and LOL fish were transplanted from the ancestral GH population one year (approximately three to four generations) ago into the two low‐predation tributaries that initially contained no resident guppies (for more information on the introduction see Travis et al. 2014). Reverse introductions from low‐predation into high‐predation sites have been attempted but unfortunately are never successful. All three low‐predation sites used in the study are bordered on both sides by barrier waterfalls that exclude all major predators. The collected juvenile guppies from all four populations were then reared under common‐garden laboratory conditions for two generations following methods explained in Gordon et al. (2015). Specifically, once fish that were collected attained adulthood, they were randomly mated and their offspring reared to form the first generation. Randomly selected first‐generation offspring from these matings were themselves then randomly mated to an unrelated male within their population to make the second generation.

Second‐generation mature adult males and females (all approximately 1 year of age) were set up in two‐gallon tanks with a 12L:12D light cycle in our laboratory in Riverside. Controlling for relatedness, one male and one female were each randomly assigned to a tank. They were then each subject to a prolonged swimming performance test largely following methods explained in Oufiero and Garland (2009) and detailed below. Females within each tank became pregnant and produced between two and three litters of offspring during the duration of the entire experiment (females store sperm and can continuously give birth to litters of offspring every 25 days; Houde 1997). All female fish were tested at least three times corresponding to three swim types carried out in consecutive order: a swim directly after given birth (1–2 days, AP), a mid‐pregnancy swim (approximately 15 days into pregnancy, MP), and a swim during the last days of pregnancy, directly before giving birth (20–24 days, VP). Most females were swum six times (three per litter) except those that had three litters and were swum nine times. All male fish were only swum twice with at least a 2 weeks gap between both swims. Fish were normally tested at similar times each day, and tests were staggered so as to standardize the time differences in swim across populations. Directly before every test, each fish was fast for 24 h, then anesthetized, weighed, and photographed. The length of each fish was later measured using the digital photographs taken next to a ruler, and the program Image J. Partial water changes were performed approximately every 2 weeks, and fish were fed flake food two times per day. In total, we used 55 males and 60 females (9 Guanapo high‐predation (GH) males, 10 Guanapo high‐predation (GH) females, 9 Guanapo low‐predation (GL) males, 10 Guanapo low‐predation (GL) females, 20 Upper Lalaja (UPL) males, 20 Upper Lalaja (UPL) females, 17 Lower Lalaja (LOL) males, 20 Lower Lalaja (LOL) females).

### Measurement of swim performance

The most common measure of prolonged or sustained swimming performance is critical swimming speed or *U*_crit_, which is the maximum velocity that can be maintained by an organism before fatigue. Our method to measure critical swimming speed (*U*_crit_) was adopted from Brett (1964) and Beamish (1978), as well as other studies Plaut (2001) and Oufiero and Garland (2009). For calculations, *U*_crit_ = *u*_i_ + (*t*_i_/*t*_ii_ × *u*_ii_), where *u*_i_ represents the highest velocity maintained by each fish (cm/sec), *u*_ii_ the velocity increments, *t*_i_ the time (min) that the fish swam at the fatigue velocity, and *t*_ii_ the total period of swimming (Brett 1964). In guppies, it has previously been shown that this method for measuring *U*_crit_ is repeatable for individual guppies, indicating that it is a good measure of swimming performance (Oufiero and Garland 2009). In this experiment, we used the same incremental velocity swim tunnel used in (Oufiero and Garland 2009). The flow tunnel held approximately 55 L of water and measured 119.5 cm (L) × 15.3 cm (W) × 18.3 cm (H) (Fig. 1). Swim performances were tested in a small arena of the swim tunnel with a plastic grating measuring 12 cm (L) × 15.3 cm (W) × 11.5 cm (H), a suitable size for smaller fish such as guppies. Temperature remained standardized at approximately 25°C throughout the experiment. Fish were acclimatized to the flow tunnel for 10 min at a low velocity (4.39 cm/sec), after which water flow was increased to 13.6 cm/sec and an addition of 3.6 cm/sec for every 3 min passed. The flow velocity in the tunnel was calibrated by filming a piece of neutrally buoyant tank‐cleaning cotton three times per speed on the swim tunnel controller. We then averaged the velocity per speed increment and used a regression to calculate the actual speed (as also described in Oufiero 2011). Every fish was tested until they could no longer maintain their position in the arena, and could not remove themselves from the back grating after two or three taps on the side of the tunnel. Time step increases for *U*_crit_ have traditionally been set at 20 min, however recently, and for smaller organism, shorter times have been used to cater to a variety of species (Breen et al. 2004; Tierney 2011). What is more critical is that multiple steps must be measured prior to fatigue.

### Statistical analysis

We used linear mixed effects models to analyze differences in prolonged swim endurance. In all models, *U*_crit_ values were used as the response variable, and individual identity was included as a random effect to account for multiple swim measurements per individual. Because performance and physiological measures often scale allometrically with size, *U*_crit_ and fish length were included as log‐transformed response and explanatory variables, respectively. In all analyses, swim times of less than three minutes were omitted, as they were considered a trial failure (e.g., due to loss of motivation). All models were fit using function ‘lme’ in package ‘nlme’, Program R v2.15 (R Core Team 2013).

First, we tested for overall sex differences in swimming performance by adding sex as an explanatory factor to the above‐mentioned mixed‐model structure.

Second, we analyzed the effect of pregnancy on females by including, as explanatory factors, pregnancy stage (AP, MP, and VP) and population (GH, GL, LOL, and UPL). Interactions between the two were not significant and therefore removed.

Third, we fit two models of differences in prolonged swimming among the populations. The first model involved only the natural GH versus GL population differences (including both sexes) to examine the effect of historical adaptation to predation on the genetic basis of prolonged swimming ability. The second involved sex‐specific differences among all population (GH, GL, LOL, UPL) to explore the influence of historical adaptation versus recent adaptation history to predation as explained previously. In both models, population of origin was included as an explanatory fixed factor, as well as its interaction with log‐length to test for differences in allometric scaling among populations, but removed if not significant.

## Results

### Effect of gender and pregnancy

In general, males had much lower average prolonged swimming (*U*_crit_) values than females regardless of population (35.904 ± 0.796 compared to 31.347 ± 0.764), and this was significant (−8.34 ± 3.236 *t* = −2.577, *P* = 0.011).

**Figure 2:**
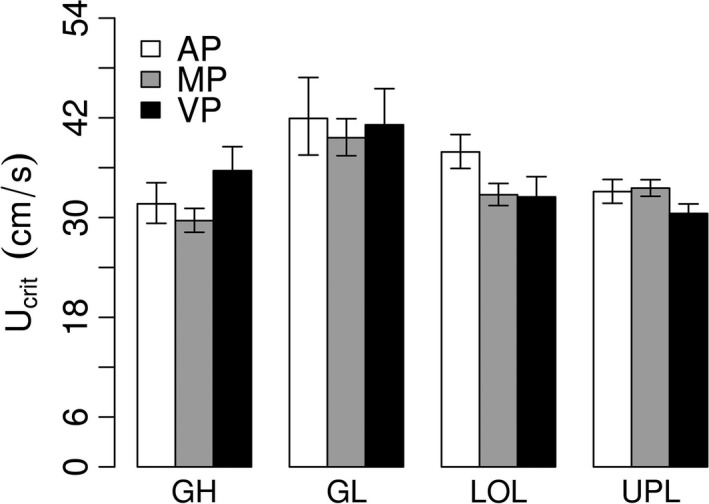
Swim endurance (*U*_crit_) differences between all four guppy populations (ancestral high‐predation GH, natural low‐predation GL, and introduced low‐predation LOL and UPL) showing separate pregnancy stages: nonpregnant fish (AP), mid‐pregnancy (MP), and latter‐stage pregnant fish (VP). Bars indicate standard errors.

Within females, there were no significant interactions between pregnancy stage, length, and population so those interactions were removed from analyses. The simplified model shows that the average critical swimming speed (*U*_crit_) of females who just had their litter (AP fish can be considered ‘nonpregnant’) was significantly higher than either females that were very pregnant (AP vs. VP: effect = 0.129 ± 0.063, *t*_141_ = 2.038, *P* = 0.043) or fish that were mid‐pregnant (AP vs. MP: effect = 0.116 ± 0.047, *t*_141_ = 2.471, *P* = 0.015; Fig. 2). This suggests a strong effect of pregnancy on swimming performance in female fish. However, unexpectedly fish that were just about to give birth (VP) did not differ from fish that were only mid‐pregnant (MP) (VP vs. MP: effect = −0.013 ± 0.058, *t*_141_ = −0.223, *P* = 0.824), suggesting that even small levels of pregnancy can strongly affect swimming performances.

### Sex‐specific population differences on swimming performance

**Figure 3:**
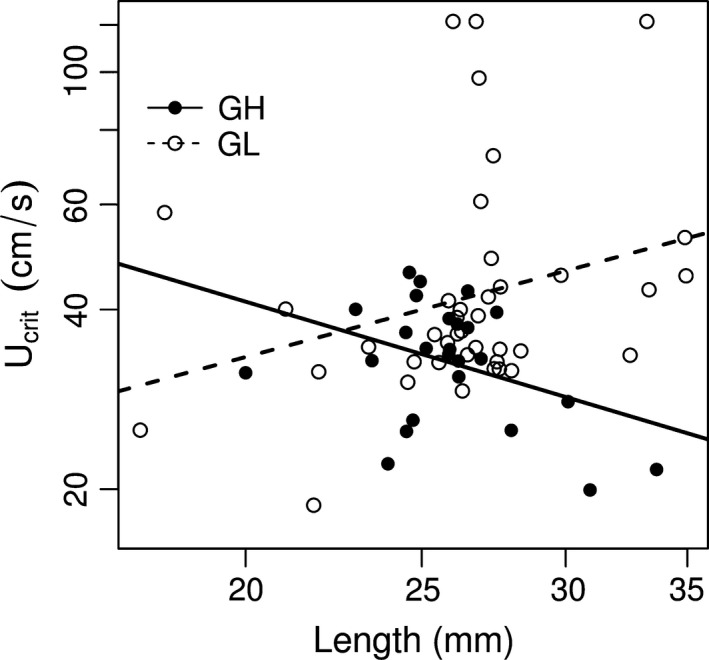
Relationship between swim endurance (*U*_crit_) and body length (mm) in females of Guanapo high‐predation GH (black circles) and Guanapo low‐predation GL (open circles) populations.

Our results show that GL (Guanapo low predation) fish have higher prolonged swimming performance or critical swimming speeds (higher *U*_crit_) than their counterpart GH (Guanapo high predation) fish (Table 1). Our use of common‐garden reared fish suggests that this difference has a genetic basis. However, this result was only significant in females and not in males. A significant interaction between body length and swimming performance indicated that this difference in females was mainly driven by differences in the *U*_crit_ of larger individuals, which performed better in GL than in GH fish, (Table 1). More specifically, in the GL, but not in the GH, larger females have longer endurance swimming performances. This is not particularly unexpected because an increase in body length generally means covering greater area with less tail‐beats, and an interaction between length and swimming performance has been previously discovered in other fish systems (Beamish 1978; Breen et al. 2004). Figure 3 shows that the relative influence of size with respect to critical swimming speed increases with increasing length in females. The distribution of lengths of individuals tested was representative in both populations (GH vs. GL: effect = 0.004 ± 0.044, *t*_18_ = −0.095, *P* = 0.926), meaning that this interaction was not solely a spurious effect of size distribution differences.

**Table 1:** LMM of log‐*U*_crit_ from natural populations as a function of population (GH vs. GL), sex and log‐length

	Value	SE	df	*T*‐value	*P*‐value
Fixed Effects					
(Intercept)	3.838	0.956	67	4.014	0.0002
Log‐length	−0.109	0.296	67	−0.370	0.712
Population (GL)	−1.760	0.847	19	−2.079	0.051
Sex (male)	−0.078	0.134	67	−0.585	0.560
Log‐length × population (GL)	0.614	0.269	67	2.285	0.025
Random effects					
Individual	0.111				
Residual	0.290				

**Figure 4:**
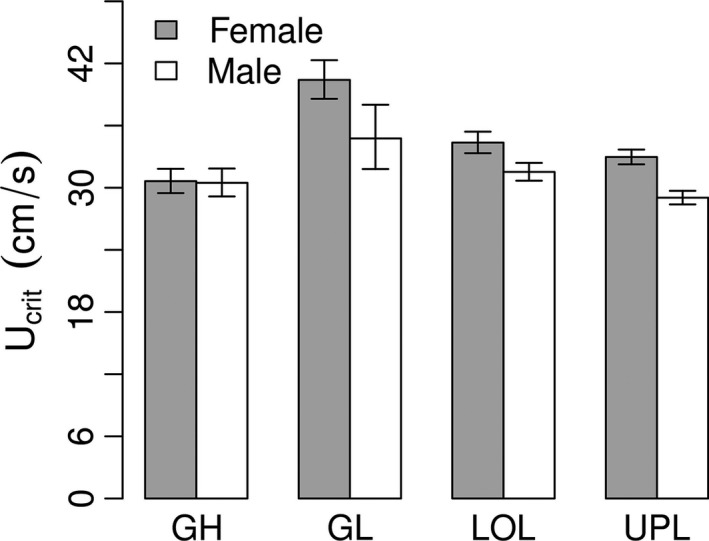
Sex‐specific population differences in swim endurance (*U*_crit_) between natural high‐predation ancestral Guanapo high‐predation fish (GH), natural Guanapo low‐predation fish (GL), and introduced low‐predation Lower and Upper Lalaja (LOL and UPL) fish. Gray columns correspond to females and white columns to males. Bars indicate standard errors.

When all four populations are included, results show that neither of the two introduced female populations (LOL, UPL) show a significant difference from their female Guanapo high‐predation (GH) ancestors (Fig. 4, GH vs. UPL: effect = −1.081 ± 2.143, *t*_56_ = −0.504532, *P* = 0.6159 and GH vs. LOL: effect = −2.428245 ± 2.229, *t*_56_ = −1.089321, *P* = 0.2807). In fact, the only significant difference (other than that between GH and GL) is between one introduction pair and the natural low‐predation population (GL vs. UPL: effect = 3.854 ± 1.328, *t*_56_ = 2.903, *P* = 0.005). This suggests that the other introduction LOL population has already diverged enough that it is now no longer significantly different from the natural low‐predation population (GL vs. LOL: effect = 2.508 ± 1.463, *t*_56_ = 1.714, *P* = 0.092). Somewhat similar results were found for males where regardless of recent historical origin or local environment neither introduced population had diverged from their ancestor (GH vs. UPL: effect = 0.846 ± 1.208, *t*_50_ = 0.700, *P* = 0.487 and GH vs. LOL: effect = −0.472 ± 1.402, *t*_50_ = −0.337, *P* = 0.738).

## Discussion

In this experiment, we have examined whether guppy populations show sex‐specific genetic differences in an important aspect of locomotive performance, swim endurance. We did this by measuring the critical swimming speed (*U*_crit_) in lab‐reared male and female guppies from populations subject to different predation pressures. Our findings can be summarized in four key results. First, we find that female guppies have significantly higher endurance values than male guppies, both within and across populations. Second, we show that as expected from theory, pregnancy (in both mid‐stage and late stage) is a disadvantage in endurance swimming, causing significantly lowered values. Third, our results suggest that predation levels do affect prolonged swimming endurance, but only in females and not in males. Specifically, female guppies from the natural high‐predation stream (Guanapo high predation = GH) have lower endurance than female fish from the natural low‐predation stream (Guanapo low predation = GL) (Fig. 4). Interestingly, gender differences in endurance are only apparent in the low‐predation environment as hypothesized in our introduction, which is consistent with more divergent selection pressures between sexes (as, e.g., would be expected with an increase in sexual selection).

Finally, we show that two populations introduced from high‐ to low‐predation sites did not show higher endurance than their high‐predation ancestors. These results suggest that adaptation may not have occurred in the short span of time and that at this stage, phylogenetic inertia weights more than recent predation shifts. We next further discuss each of these findings in greater details.

### Effect of gender

There are a few ideas to explain why female guppies may benefit from higher endurance than males, especially in the low‐predation environment. First, our results could be explained in light of locomotive trade‐offs between burst swimming and endurance. Prior research has already shown that rapid escape abilities or burst swimming is higher in both males and females in high‐predation environments compared to low‐predation ones (O'Steen et al. 2002; Ghalambor et al. 2003) and that endurance swimming is generally higher in low‐predation male guppies compared to high‐predation ones (Nicoletto and Kodric‐Brown 1999). Since females experience different selective pressures than males within each environment, they may invest differently in endurance swimming than burst swimming in comparison with males within those environments. For example, female guppies are larger and tan in coloration, but male guppies are colorful, more noticeable to predators, and hence have consistently been shown to have much higher mortality rates, in both environment types (Reznick et al. 1996; Magurran 2005; Gordon et al. 2009). Perhaps, males have lower overall endurance levels than females because, given this higher mortality cost, they have invested more in the swimming type that will enable them to better escape predation attempts.

A second reason that need not be mutually exclusive may be that males (who have determinate growth) forage considerably less than females (with indeterminate growth) during adulthood and instead are more focused on mate searching. While adult females often drift‐feed on the faster‐flowing end of pools riffles (where they may need greater endurance levels for efficient foraging), males are more often found in the slower flowing pool areas of the stream preoccupied with easier mating opportunities (Marshall et al. 2012).

### Effect of pregnancy

As expected, we found an effect of pregnancy on prolonged swimming endurance in females. Pregnancy has important ecological and evolutionary consequences for female swim performance (Svendsen et al. 2013), yet is rarely studied to that effect. In a variety of organisms, pregnancy or gravidity is associated with increased metabolic rates or costs (Birchard et al. 1984; DeMarco 1993; Timmerman and Chapman 2003), shifting morphological forms, and reduced locomotive ability (Magnhagen 1991; Plaut 2002; Ghalambor et al. 2004; Webb and Lannoo 2004). Females have to invest much of their energy into reproduction (producing and carrying eggs), which should affect their swimming performance (Plaut 2002; Colborne et al. 2011), especially when predators are present (Ghalambor et al. 2004). For example, Plaut (2002) found reduced endurance performance in pregnant compared to nonpregnant mosquito fish (*Gambusia affinis*), likely caused by increased energy costs and warped body form. These effects are generally more pronounced as pregnancy progresses.

Our study did find an expected effect of pregnancy on swimming endurance, with females showing lower endurance when pregnant than when tested soon after giving birth. In guppies, as well as many other fish, pregnancy, in addition to causing a greater energetic burden, also results in a large distension of the body, which results in a deeper body. Previous work using various fish systems shows that deeper bodies results in slower prolonged/sustained swimming due to increased drag (Webb 1994). Indeed, a study by Ghalambor et al. (2004) indicated a functional trade‐off between burst swimming performance and increasing pregnancy in guppies. It showed that female guppies from high‐predation localities had better performances in burst swimming, but that over the course of pregnancy, this performance declined. Unlike the Ghalambor et al. (2004) study, we detected no significant reductions in endurance as pregnancy progressed from middle to latter stages of pregnancy. These results may be an artifact of swim type, such that the likely cost of frictional drag associated with the deeper bodies in late‐stage pregnancy might be offset by the benefits deep bodies provide in the reduction of recoil in endurance swim (Webb 1992). As a result, morphologically speaking, the deeper body at late pregnancy may play a weaker role in affecting swim endurance performance than it would other aspects of swim such as burst swim. In order to better examine this, studies on a greater number of populations and species are needed. In the meanwhile, we hope our study fills a needed gap (according to Svendsen et al. 2013) by further examining the effect of pregnancy (and potentially predation risk) on swim endurance using common‐garden fish.

### Sex‐specific population differences on swimming performance

Predation can drive organisms to adopt more vigorous escape behaviors or faster burst swimming (mammals: Gebo et al. 1994; Taraborelli et al. 2003; reptiles: Irschick and Losos 1998; amphibians: Watkins 1994; fish: O'Steen et al. 2002; Domenici et al. 2008). On the other hand, environments lacking predators may select for increased prolonged or sustained swimming; strategies that are instead better for finding food or mates (Oufiero et al. 2011). As a result, abrupt environmental shifts in predation levels could influence changes in swimming performance due to changes in selection pressures. This could occur via direct (e.g., increasing burst speed in response to increase in predation) or indirect means (e.g., losing sustained swimming due to trade‐off with burst swim in response to the increase in predation) (Dalziel et al. 2011). Therefore, the evolution of swimming performance is directly and indirectly related to the combination of selective factors acting on a suite of different traits (Ghalambor et al. 2003), and this can have a sex‐specific effect.

Our results for females are consistent with the above theory, with low‐predation females showing higher endurance (Fig. 4). Males did not show a clear population effect. This could be due to the fact that, again, while females might benefit from increased endurance foraging in fast open waters with few predators, males spend a lower proportion of time foraging and more in courtship and mate competition. Despite this, a previous study examining prolonged endurance swimming in wild guppy males showed increased endurance in low‐predation populations compared to high‐predation ones (Nicoletto and Kodric‐Brown 1999). However, it should be noted that their Guanapo LP males showed the smallest and insignificant increase in endurance compared to their HP ancestors (Nicoletto and Kodric‐Brown 1999). This means that our results for lab‐reared males in this study have repeated what the previous study found using wild males for this particular river. This does highlight one limitation of our study, which is that our high‐ versus low‐predation contrasts are only performed in one river, due to rearing logistics, and hence, any conclusions drawn should reflect this limitation. Nevertheless, our study is the first, to our knowledge, to examine the genetic basis of prolonged endurance swimming in a rapidly evolving system, using common garden rather than wild fish. We therefore hope this will spur more studies examining whether the here‐observed differences in swimming endurance are consistently related to predation.

Second, our results show that neither of the two transplanted populations showed a significant difference in endurance from their high‐predation ancestor, despite the fact that we have already found rapid evolution of body size and other traits in these fish (Travis et al. 2014; Gordon et al. 2015). One of the streams, however (LOL), did show a large trend in the expected direction and is also not significantly different from the natural low‐predation (GL) population. This suggests that it may be only a matter of time before we begin to see significant differences in swim between the ancestral and introduced populations. Therefore, overall these results are inconclusive with regard to the possibility of rapid evolutionary adaptation of endurance to low‐predation environments, in a species known for the rapid evolution of many of its morphological, physiological, and life‐history traits (Endler 1980; Gordon et al. 2009). Instead so far, they seem to point toward translocated populations still bearing a strong signature of the historical adaptation to their ancestral high‐predation regime. It will be interesting to see how these results change given more time.

## Conclusion

Swim endurance in fish is an example of a fitness‐related whole‐organism performance trait (Dalziel et al. 2011) involving multiple interactions between various traits. Here, we have shown sex‐specific genetic differences in prolonged swimming performance between populations differing in predation pressure and thus the potential to evolve in response to selection. However, its response to abrupt changes in the environment may lag behind other morphological traits known to evolve rapidly in these populations.

## Conflict of Interest

None declared.

## References

[ece31789-bib-0001] Alexander, H. J. , J. S. Taylor , S. S.‐T. Wu , and F. Breden . 2006 Parallel evolution and vicariance in the guppy (*Poecilia reticulata*) over multiple spatial and temporal scales. Evolution 60:2352–2369. 17236426

[ece31789-bib-0002] Aubret, F. , X. Bonnet , and R. Shine . 2007 The role of adaptive plasticity in a major evolutionary transition: early aquatic experience affects locomotor performance of terrestrial snakes. Funct. Ecol. 21:1154–1161. doi:10.1111/j.1365-2435.2007.01310.x.

[ece31789-bib-0003] Basolo, A. L. , and G. Alcaraz‐Zubeldia . 2003 The turn of the sword: energetic costs of a sexually selected trait. Proc. R. Soc. Lond. B 270:1631–1636.

[ece31789-bib-0004] Beamish, F. W. H. 1978 Swimming capacity Pp. 101–187 *in* Hoar W. S. and Randall D. J., eds. Fish physiology. Academic Press, New York, NY.

[ece31789-bib-0005] Birchard, G. F. , C. P. Black , G. Schuett , and V. Black . 1984 Influence of pregnancy on oxygen consumption, heart rate, and hematology in the garter snake: implications for the “cost of reproduction” in live bearing reptiles. Comp. Biochem. Physiol. 77:519–523.

[ece31789-bib-0006] Bisazza, A. 1993 Male competition, female mate choice and sexual size dimorphism in poeciliid fishes. Mar. Behav. Physiol. 23:257–286.

[ece31789-bib-0007] Blake, R. W. 2004 Fish functional design and swimming performance. J. Fish Biol. 65:1193–1222. 10.1111/j.1095-8649.2009.02309.x 20738559

[ece31789-bib-0008] Breen, M. , J. Dyson , F. G. Oneill , E. Jones , and M. Haigh . 2004 Swimming endurance of haddock (*Melanogrammus aeglefinus* L.) at prolonged and sustained swimming speeds, and its role in their capture by towed fishing gears. ICES J. Mar. Sci. 61:1071–1079.

[ece31789-bib-0009] Brett, J. R. 1964 The respiratory metabolism and swimming performance of young sockeye salmon. J. Fish. Res. Bd. Canada 21:1183–1226.

[ece31789-bib-0010] Colborne, S. F. , M. C. Bellemare , P. R. Peres‐Neto , and B. D. Neff . 2011 Morphological and swim performance variation among reproductive tactics of bluegill sunfish (*Lepomis macrochirus*). Can. J. Fish Aquat. Sci. 68:1802–1810.

[ece31789-bib-0011] Dalziel, A. C. , T. H. Vines , and P. M. Schule . 2011 Reductions in prolonged swimming capacity following freshwater colonization in multiple threespine stickback populations. Evolution 66:1226–1239. 22486700 10.1111/j.1558-5646.2011.01498.x

[ece31789-bib-0012] DeMarco, V. 1993 Metabolic rates of female viviparous lizards (*Sceloporus jarrovi*) throughout the reproductive cycle: do pregnant lizards adhere to standard allometry? Physiol. Zool. 66:166–180.

[ece31789-bib-0013] Domenici, P. G. 2003 Habitat, body design and the swimming performance of fish Pp. 137–160 *in* Bels V. L., Gasc J. P. and Casinos A., eds. Vertebrate biomechanics and evolution and evolution. BIOS Scientific Publishers Ltd, Oxford.

[ece31789-bib-0014] Domenici, P. G. , and R. W. Blake . 1997 The kinematics and performance of fish fast‐start swimming. J. Exp. Biol. 200:1165–1178. 9319004 10.1242/jeb.200.8.1165

[ece31789-bib-0015] Domenici, P. G. , H. Turesson , J. Brodersen , and C. Bronmark . 2008 Predator induced morphology enhances escape locomotion in crucian carp. Proc. R. Soc. Lond. Ser. B 275:195–201. 10.1098/rspb.2007.1088 PMC2596180 17971327

[ece31789-bib-0016] Endler, J. A. 1980 Natural selection on color patterns in *Poecilia reticulata* . Evolution 34:76–91. 28563214 10.1111/j.1558-5646.1980.tb04790.x

[ece31789-bib-0017] Fulton, C. J. , D. R. Bellwood , and P. C. Wainwright . 2005 Wave and swimming performance shape coral reef fish assemblages. Proc. R. Soc. Lond. Ser. B 272:827–832. 10.1098/rspb.2004.3029 PMC1599856 15888415

[ece31789-bib-0018] Gebo, D. L. , C. A. Chapman , L. J. Chapman , and J. Lambert . 1994 Locomotor responses to predator threat in red colobus monkeys. Primates 35:219–223.

[ece31789-bib-0019] Ghalambor, C. K. , J. A. Walker , and D. N. Reznick . 2003 Multi‐trait selection, adaptation, and constraints on the evolution of burst swimming performance. Integr. Comp. Biol. 43:431–438. 21680451 10.1093/icb/43.3.431

[ece31789-bib-0020] Ghalambor, C. K. , D. N. Reznick , and J. A. Walker . 2004 Constraints on adaptive evolution: the functional trade‐off between reproduction and fast‐start swimming performance in the Trinidadian guppy (*Poecilia reticulata*). Am. Nat. 164:38–50. 15266369 10.1086/421412

[ece31789-bib-0021] Gordon, S. P. , D. N. Reznick , M. T. Kinnison , M. J. Bryant , D. J. Weese , K. Räsänen , et al. 2009 Adaptive changes in life history and survival following a new guppy introduction. Am. Nat. 174:34–45. 19438322 10.1086/599300

[ece31789-bib-0022] Gordon, S. P. , D. N. Reznick , J. Arendt , A. Roughton , H. M. Ontiveros , P. Bentzen , et al. 2015 Selection analysis on the rapid evolution of a secondary sexual trait. Proc. R. Soc. Lond. B 282:20151244. 10.1098/rspb.2015.1244 PMC4632628 26290077

[ece31789-bib-0023] Hendry, A. P. , M. L. Kelly , M. T. Kinnison , and D. N. Reznick . 2006 Parallel evolution of sexes? Effects of predation and habitat features on the size and shape of wild guppies. Evol. Biol. 19:741–754. 10.1111/j.1420-9101.2005.01061.x 16674571

[ece31789-bib-0024] Higham, T. E. 2007 The integration of locomotion and prey capture in vertebrates: morphology, behavior, and performance. Integr. Comp. Biol. 47:82–91. 21672822 10.1093/icb/icm021

[ece31789-bib-0025] Houde, A. E. 1997 Sex, color and mate choice in guppies. Princeton Univ. Press, Princeton, NJ.

[ece31789-bib-0026] Huntingford, F. A. , P. J. Wright , and J. F. Tierney . 1994 Adaptive variation in antipredator behaviour in threespine stickleback Pp. 277–296 *in* Bell M. A. and Foster S. A., eds. The evolutionary biology of the Threespine stickleback. Oxford Univ. Press, Oxford, U.K.

[ece31789-bib-0027] Irschick, D. J. , and J. B. Losos . 1998 A comparative analysis of the ecological significance of locomotor performance in Caribbean *Anolis* lizards. Evolution 52:219–226. 28568148 10.1111/j.1558-5646.1998.tb05155.x

[ece31789-bib-0028] Langerhans, R. B. 2009 Trade‐off between steady and unsteady swimming underlies predator‐driven divergence in *Gambusia affinis* . J. Evol. Biol. 22:1057–1075. 21462405 10.1111/j.1420-9101.2009.01716.x

[ece31789-bib-0029] Langerhans, R. B. , and T. J. DeWitt . 2004 Shared and unique features of evolutionary diversification. Am. Nat. 164:335–349. 15478089 10.1086/422857

[ece31789-bib-0030] Langerhans, R. B. , and D. N. Reznick . 2010 Ecology and Evolution of Swimming Performance in Fishes: predicting Evolution with Biomechanics Pp. 200–248 *in* Kapoor B. G. and Domenici P., eds. Fish locomotion: an eco‐ethological perspective. Science Publishers, Oxford.

[ece31789-bib-0031] Langerhans, R. B. , C. A. Layman , A. M. Shokrollahi , and T. J. DeWitt . 2004 Predator driven phenotypic diversification in *Gambusia affinis* . Evolution 58:2305–2318. 15562692 10.1111/j.0014-3820.2004.tb01605.x

[ece31789-bib-0033] Magnhagen, C. 1991 Predation risk as a cost of reproduction. Trends Ecol. Evol. 6:183–186. 21232452 10.1016/0169-5347(91)90210-O

[ece31789-bib-0034] Magurran, A. E. 2005 Evolutionary ecology: the Trinidadian guppy. Oxford Univ. Press, New York.

[ece31789-bib-0035] Marras, S. , S. Killen , G. Claireaux , P. G. Domenici , and D. McKenzie . 2011 Behavioral and kinematic components of the fast‐start escape response in fish: individual variation and temporal repeatability. J. Exp. Biol. 214:3102–3110. 21865523 10.1242/jeb.056648

[ece31789-bib-0036] Marshall, M. C. , A. J. Binderup , E. Zandonà , S. Goutte , R. D. Bassar , R. W. El‐Sabaawi , et al. 2012 Effects of consumer interactions on benthic resources and ecosystem processes in a Neotropical stream. PLoS One 7:e45230. 23028865 10.1371/journal.pone.0045230 PMC3461008

[ece31789-bib-0037] McGuigan, K. , C. E. Franklin , C. Moritz , and M. W. Blows . 2003 Adaptation of rainbowfish to lake and stream habitat. Evolution 57:104–118. 12643571 10.1111/j.0014-3820.2003.tb00219.x

[ece31789-bib-0038] Nicoletto, P. F. , and A. Kodric‐Brown . 1999 The relationship among swimming performance, courtship behavior, and carotenoid pigmentation of guppies in four rivers in Trinidad. Environ. Biol. Fish. 55:227–235.

[ece31789-bib-0039] O'Steen, S. , A. J. Cullum , and A. F. Bennett . 2002 Rapid evolution of escape ability in Trinidadian Guppies (*Poecilia reticulata*). Evolution 56:776–784. 12038535 10.1111/j.0014-3820.2002.tb01388.x

[ece31789-bib-0040] Oufiero, C. E. 2011 The Cost of Bearing a Sword: Locomotor Costs and Compensations in Relation to a Sexually Selected Trait in Xiphophorus. UCR Electronic Theses and Dissertations. http://escholarship.org/uc/item/4st8r4bf.

[ece31789-bib-0042] Oufiero, C. E. , and T. Jr Garland . 2009 Repeatability of correlation of swimming performances and size over varying time‐scales in the guppy (*Poecilia reticulata*). Funct. Ecol. 23:969–978.

[ece31789-bib-0043] Oufiero, C. E. , M. R. Walsh , D. N. Reznick , and T. Jr Garland . 2011 Swimming performance trade‐offs across a gradient in community composition in Trinidadian killifish (*Rivulus hartii*). Ecology 92:170–179. 21560687 10.1890/09-1912.1

[ece31789-bib-0044] Pigliucci, M. 2003 Phenotypic integration: studying the ecology and evolution of complex phenotypes. Ecol. Lett. 6:265–272.

[ece31789-bib-0045] Plaut, I. 2001 Critical swimming speed: its ecological relevance. Comp. Biochem. Physiol. A 131:41–50. 10.1016/s1095-6433(01)00462-7 11733165

[ece31789-bib-0046] Plaut, I. 2002 Does pregnancy affect swimming performance of female Mosquitofish, *Gambusia affinis*? Func. Ecol. 16:290–295.

[ece31789-bib-0505] R Core Team. 2013 R: A language and environment for statistical computing. R Foundation for Statistical Computing, Vienna, Austria. URL http://www.R-project.org/.

[ece31789-bib-0047] Reznick, D. N. , M. J. Butler , F. H. Rodd , and P. Ross . 1996 Life history evolution in guppies (*Poecilia reticulata*). 6. Differential mortality as a mechanism for natural selection. Evolution 50:1651–1660. 28565709 10.1111/j.1558-5646.1996.tb03937.x

[ece31789-bib-0048] Suk, H. Y. , and B. D. Neff . 2009 Microsatellite genetic differentiation among populations of the Trinidad guppy. Heredity 102:425–434. 19223925 10.1038/hdy.2009.7

[ece31789-bib-0049] Svendsen, J. C. , A. I. Banet , R. H. B. Christensen , J. F. Steffensen , and K. Aarestrup . 2013 Effects of intraspecific variation in reproductive traits, pectoral fin use and burst swimming on metabolic rates and swimming performance in the Trinidadian guppy (*Poecilia reticulata)* . J. Exp. Biol. 216:3564–3574. doi:10.1242/jeb.083089. 23737561

[ece31789-bib-0050] Taraborelli, P. , V. Corbalan , and S. M. Giannoni . 2003 Locomotion and escape modes in rodents of the Monte Desert (Argentina). Ethology 109:475–485.

[ece31789-bib-0051] Tierney, K. B. 2011 Swimming performance assessment in fishes. J. Vis. Exp. 51:e2572. doi:10.3791/2572. PMC3197426 21633333

[ece31789-bib-0052] Timmerman, C. M. , and L. J. Chapman . 2003 The effect of festational state on oxygen consumption and response to hypoxia in the sailfin molly, *Poecilia latipinna* . Environ. Biol. Fishes 68:293–299.

[ece31789-bib-0053] Travis, J. D. , D. R. Reznick , R. D. Bassar , A. López‐Sepulcre , R. Ferriere , and T. Coulson . 2014 Do eco‐evo feedbacks help us understand nature? Answers from studies of the Trinidadian guppy. Adv. Ecol. Res. 50:1–40.

[ece31789-bib-0054] Watkins, T. B. 1994 Predator‐mediated selection on burst swimming performance in tadpoles of the Pacific tree frog *Pseudaris regilla* . Physiol. Zool. 69:154–167.

[ece31789-bib-0055] Webb, P. W. 1984 Body form, locomotion, and foraging in aquatic vertebrates. Am. Zool. 24:107–120.

[ece31789-bib-0056] Webb, P. W. 1992 Is the high cost of body/caudal fin undulatory swimming due to increased friction drag or inertial recoil? J. Exp. Biol. 162:157–166.

[ece31789-bib-0057] Webb, P. W. 1994 Exercise performance of fish Pp. 1–49 *in* Jones J. H., ed. Advances in veterinary science and comparative medicine, vol. 38 B. comparative vertebrate exercise physiology: phyletic adaptations. Academic Press, San Diego, CA. 7810375

[ece31789-bib-0058] Webb, J. K. , and M. J. Lannoo . 2004 Pregnancy decreases swimming performance of female northern death adders (*Acanthophis praelongus*). Copeia 2:357–363.

[ece31789-bib-0059] Weihs, D. 1973 The mechanism of rapid starting of slender fish. J. Biorheol. 10:343–350. 10.3233/bir-1973-10308 4772008

